# Supplementary material for: Negri bodies are viral factories with properties of liquid organelles
Source: Nat Commun. 2017 Jul 5;8:58. doi: 10.1038/s41467-017-00102-9 (PMC5498545; doi:10.1038/s41467-017-00102-9)
Supplement: Supplementary file 1 — Supplementary Information [file 41467_2017_102_MOESM1_ESM.pdf]

Title of file for HTML: Supplementary Information
Description: Supplementary Figures and Supplementary Table

Title of file for HTML: Supplementary Movie 1
Description: Fusion of NBs. BSR cells were infected by rCVSN2C-P-mCherry and imaged over time using live cell microscopy. The time post infection is indicated in the lower left corner.

Title of file for HTML: Supplementary Movie 2
Description: Fusion of NBs. BSR cells were infected by rCVSN2C-P-mCherry and imaged over time using live cell microscopy. The time post infection is indicated in the lower left corner.

Title of file for HTML: Supplementary Movie 3
Description: A spherical cytoplasmic "bubble" crosses a NB. BSR cells were infected by rCVSN2C-P-mCherry and imaged over time using live cell microscopy. The time post infection is indicated in the lower left corner.

Title of file for HTML: Supplementary Movie 4
Description: NBs are sensitive to a hypotonic chock. BSR cells were infected by rCVSN2C-P-mCherry and imaged over time using live cell microscopy. A hypotonic shock was applied 5 minutes after the beginning of the movie (~18h p.i.). The time post-infection is displayed in the lower left corner.

Title of file for HTML: Supplementary Movie 5
Description: NBs and SGs are non-miscible liquid organelles. U373-MG cells were transiently transfected with pG3BP-eGFP (to visualize SGs in white). 1h post transfection, they were infected with the recombinant virus rCVSN2C-P-mCherry (to visualize NBs in red). At 16h p.i., both NBs and SGs were imaged. The time post-infection is displayed in the lower left corner.

Title of file for HTML: Supplementary Movie 6
Description: RNPs are ejected from NBs. BSR cells were infected by rCVSN2C-P-mCherry BSR cells were infected by rCVSN2C-P-mCherry. At 16h p.i., NBs and RNPs were imaged. The time post-infection is displayed in the lower left corner.

Title of file for HTML: Supplementary Movie 7
Description: RNPs are ejected from NBs. BSR cells were infected by rCVSN2C-P-mCherry BSR cells were infected by rCVSN2C-P-mCherry. At 16h p.i., NBs and RNPs were imaged. The time of acquisition is displayed in the lower left corner.

Title of file for HTML: Supplementary Movie 8
Description: RNPs are mobile in the cytosol of infected cells. BSR cells were infected by rCVSN2C-P-mCherry. At 20h p.i., NBs and RNPs were imaged. The time post-infection is displayed in the lower left corner.

Title of file for HTML: Supplementary Movie 9

Description: Nocodazole inhibits RNPs movement. Nocodazole (2 µM) was added 1h before and kept all along infection of BSR cells by rCVSN2C-P-mCherry. At ~23h p.i., NBs and RNPs were imaged. The time post-infection is displayed in the lower left corner.

Title of file for HTML: Supplementary Movie 10
Description: RNPs are transported along microtubules. BSR cells were co-infected with rCVS N2C-P-mCherry and a modified baculovirus encoding human tubulin-GFP. Cells were imaged after 16h p.i.

Title of file for HTML: Supplementary Movie 11
Description: No ejection is observed from N-P like structures in the minimal system. BSR-T7/5 cells were co-transfected with plasmids pTit-P-mCherry and pTit-N. Cells were imaged at ~24h post-transfection.

Title of file for HTML: Supplementary Movie 12
Description: No ejection is observed from N-P like structures in the minimal system (when co-tranfected with pTit M). BSR-T7/5 cells were co-transfected with plasmids pTit-P-mCherry, pTit-N and pTit-M. Cells were imaged at ~24h post-transfection.

Title of file for HTML: Peer Review File
Description:

Supplementary Information

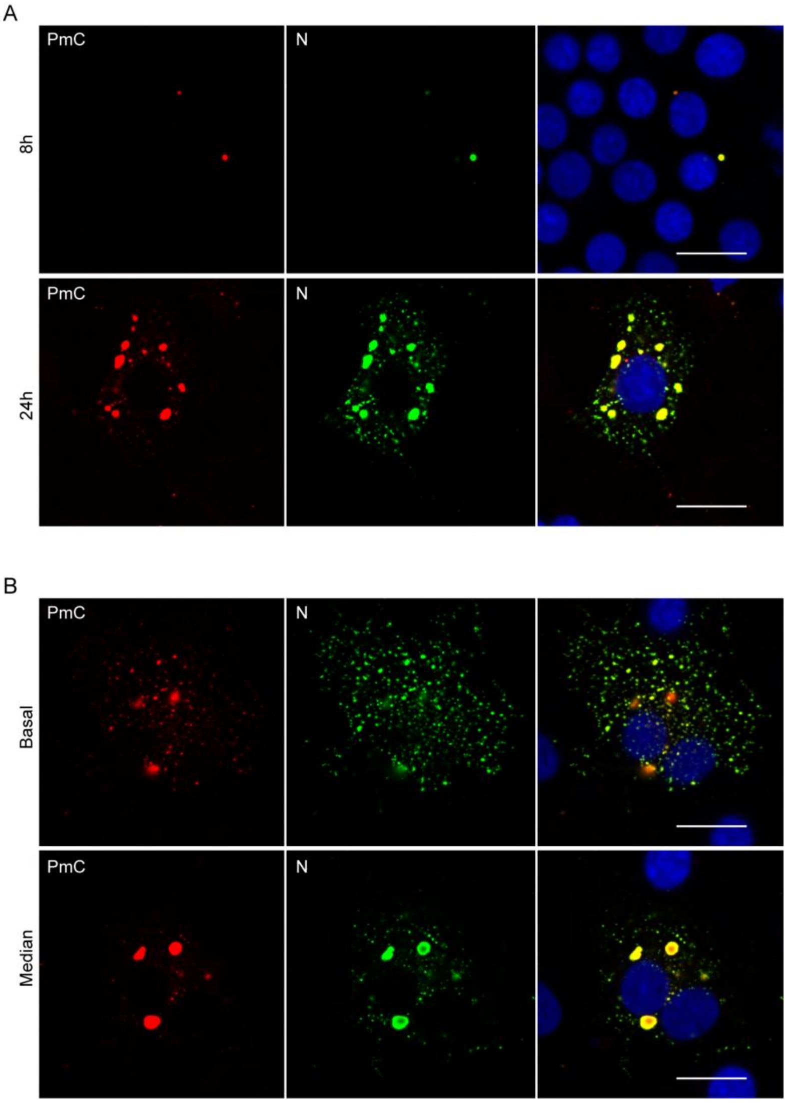

Supplementary Figure 1 (related to Figure 2): rCVSN2C-P-mCherry behaves as wild type CVS.

BSR cells were infected with rCVSN2C-P-mCherry at a MOI of 0.5 and fixed at 8 and 24h p.i.

(A) Confocal analysis was performed after staining with a mouse monoclonal anti-N antibody followed by incubation with Alexa-488 donkey anti-mouse IgG. DAPI was used to stain the nuclei.

(B) Confocal analysis revealing the basal localization of small dots and the median localization of inclusions in RABV infected cells at 24h p.i. The analysis was performed after staining as in (A).

Scale bars correspond to 15 µm.

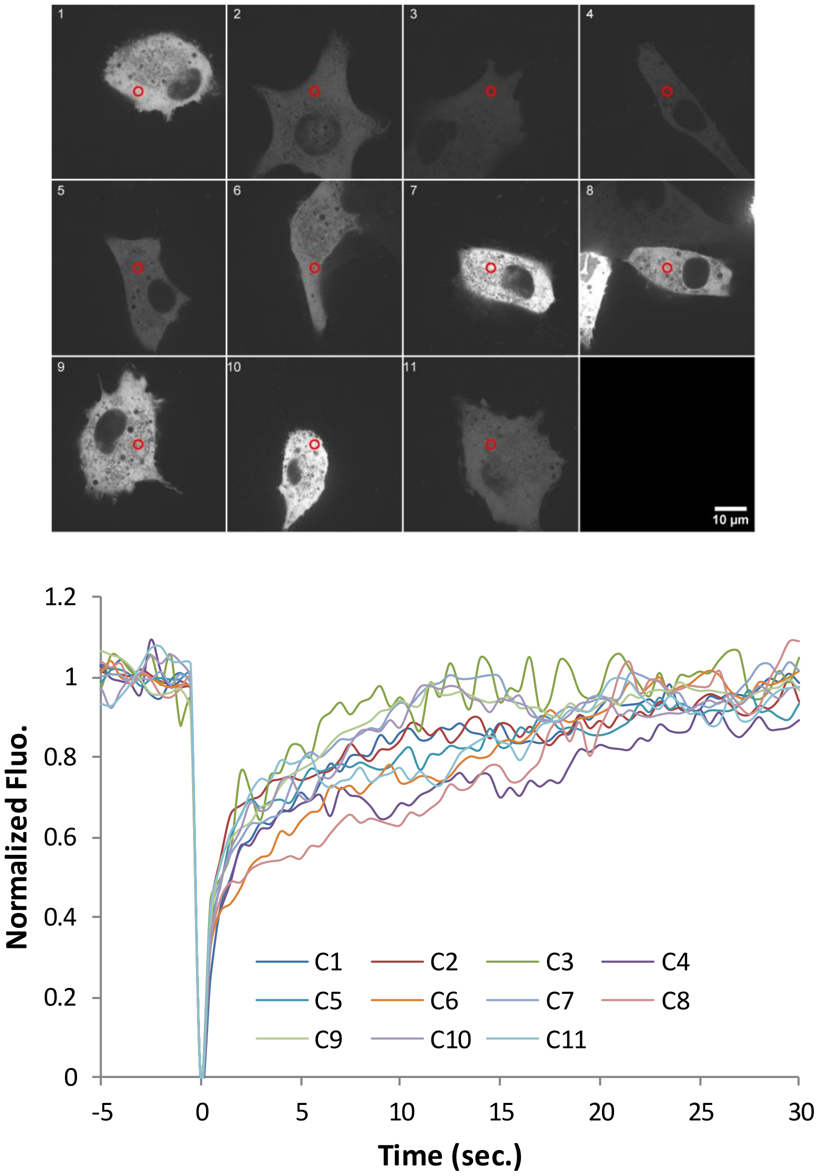

Supplementary Figure 2 (related to Figure 2E): Fluorescence recovery after photobleaching (FRAP) of P-mCherry expressed after transfection in BSR cells

Cytosolic P-mCherry was photobleached 24h after transfection of pTit-P-mCherry plasmid. The FRAP curves corresponding to each of the 11 FRAP events are shown. They have been used to obtain the mean curve of figure 2E.

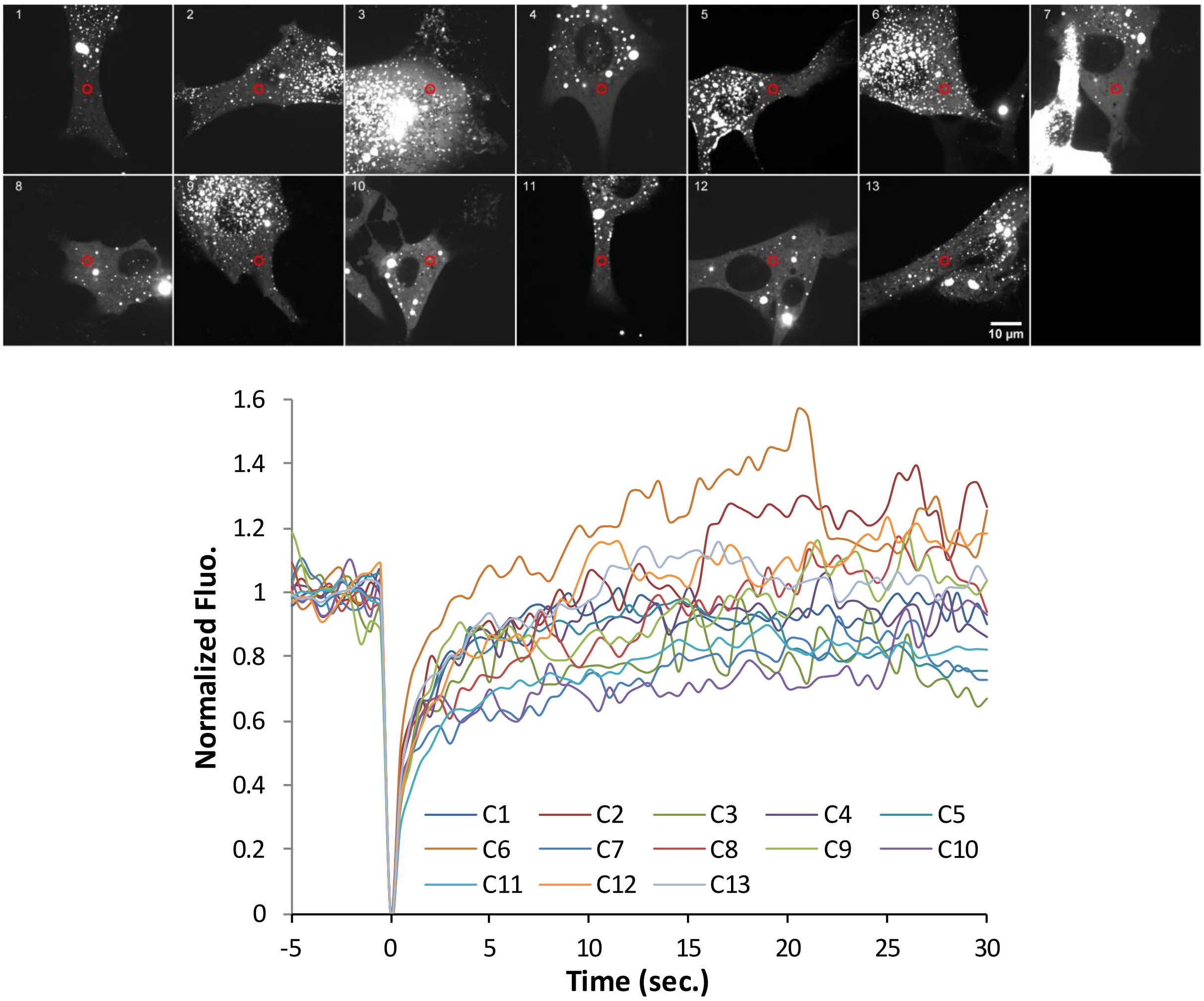

Supplementary Figure 3 (related to Figure 2F): Fluorescence recovery after photobleaching (FRAP) of cytosolic P-mCherry expressed in BSR cells infected by rCVSN2CΔG-P-mCherry.

Cytosolic P-mCherry was photobleached 16h p.i. The FRAP curves corresponding to each of the 13 FRAP events are shown. They have been used to obtain the mean curve of figure 2F.

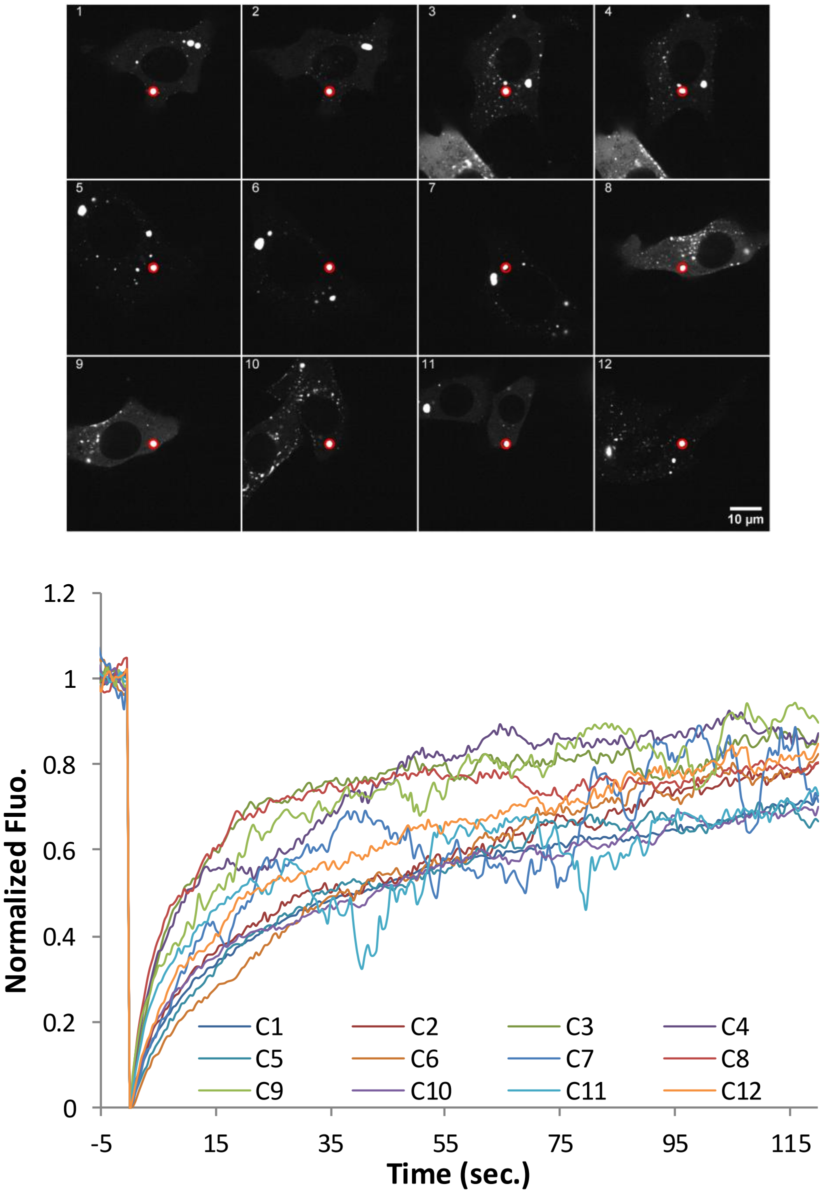

Supplementary Figure 4 (related to Figure 2G): Fluorescence recovery after photobleaching (FRAP) of P-mCherry in NBs formed in BSR cells infected by rCVSN2CΔG-P-mCherry.

Cytosolic P-mCherry located in NBs was photobleached 16h p.i. The FRAP curves corresponding to each of the 12 FRAP events are shown. They have been used to obtain the mean curve of figure 2G.

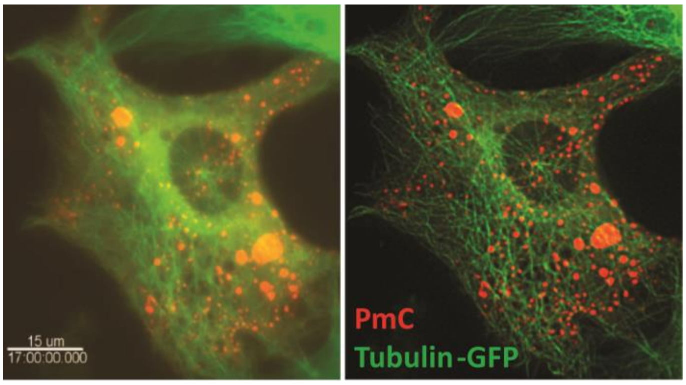

Supplementary Figure 5 (related to Figure 5): Images of BSR cells co-infected with rCVS N2C-P-mCherry and a modified baculovirus encoding human tubulin-GFP (Cell-light Tubulin-GFP) before (left) and after (right) deconvolution using the Huygens Imaging software.

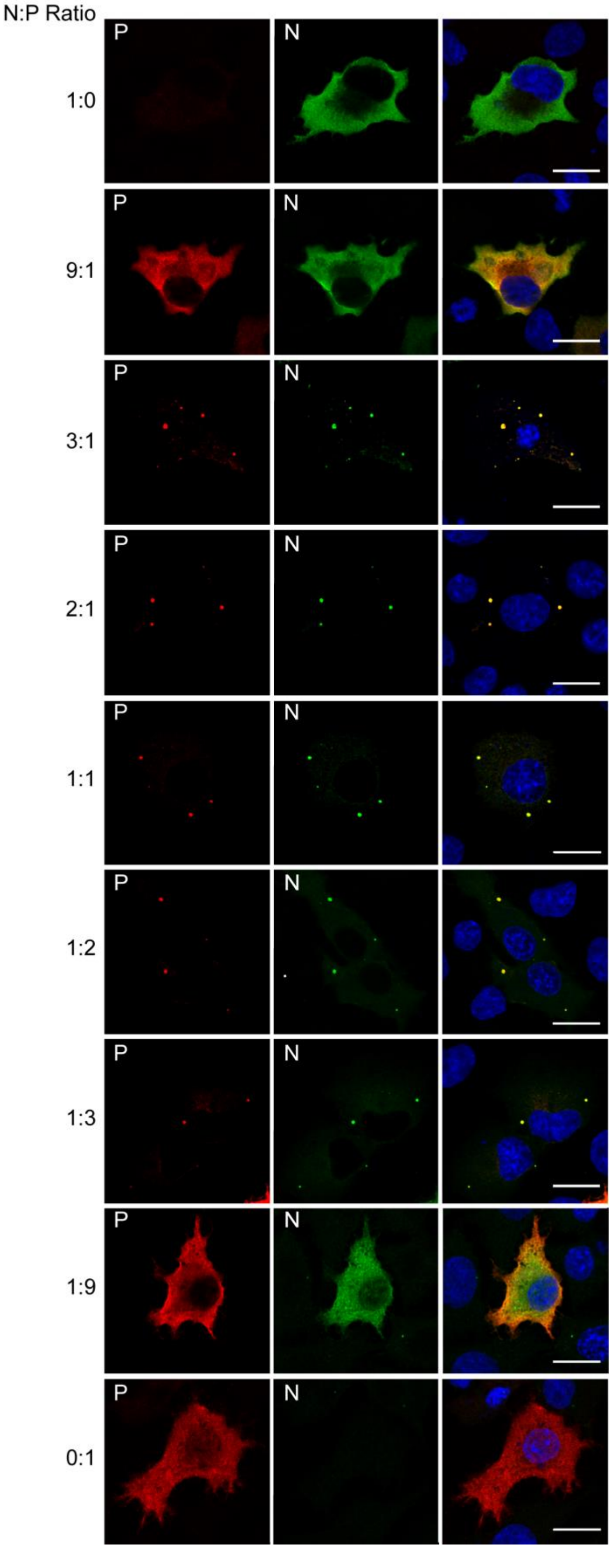

Supplementary Figure 6 (related to Figure 6A): Dependence of NB-like structures formation on the stoechiometry of pTit-N and pTit-P plasmids.

BSR-T7/5 cells were co-transfected with different ratios of pTit-P and pTit-N (keeping constant the total amount of DNA to 800ng for 3 10^5 cells). N was revealed with a mouse monoclonal anti-N antibody followed by incubation with Alexa-488 donkey anti-mouse IgG and P was revealed with a rabbit polyclonal anti-P antibody followed by incubation with Alexa-568 donkey anti-rabbit IgG. DAPI was used to stain the nuclei. Scale bars correspond to 15 µm.

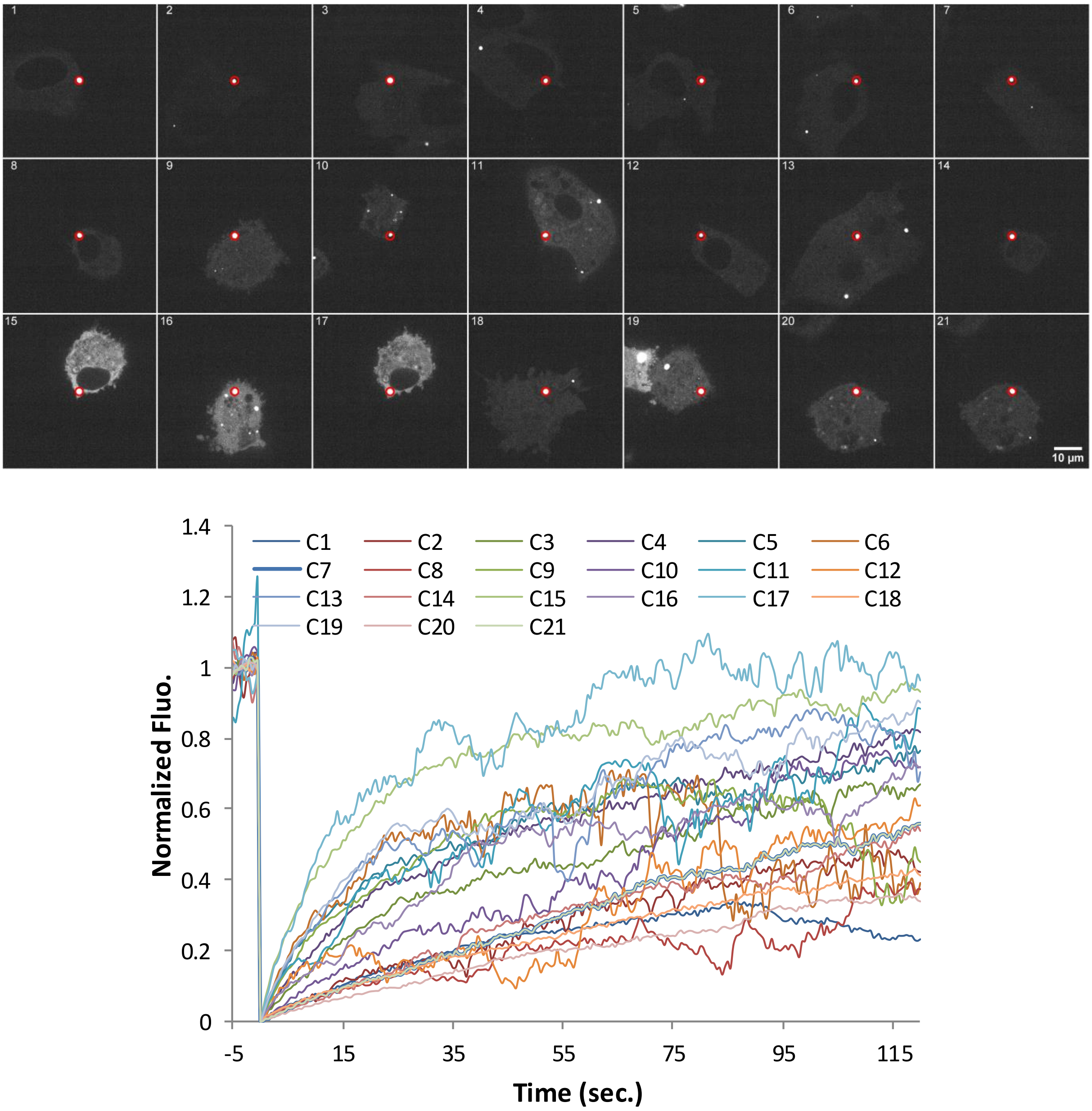

Supplementary Figure 7 (related to Figure 6B): Fluorescence recovery after photobleaching (FRAP) of P-mCherry in NB-like inclusions formed in BSR-T7/5 cells co-transfected by pTit-P-mCherry and pTit-N.

Cytosolic P-mCherry located in NB-like inclusions was photobleached 24h post-transfection. The FRAP curves corresponding to each of the 21 FRAP events are shown. They have been used to obtain the mean curve of figure 6B.

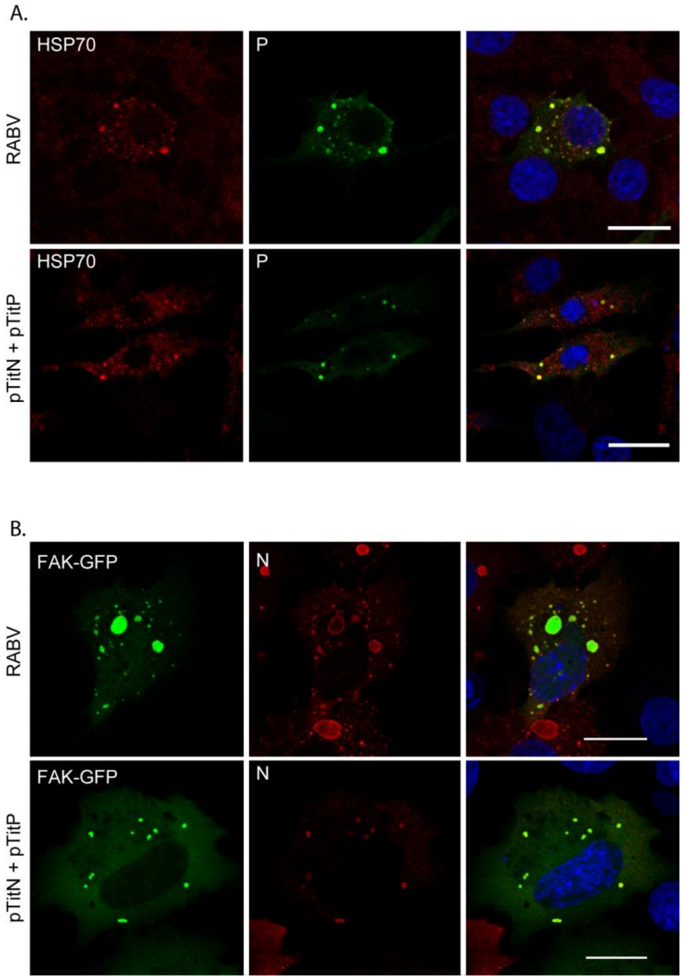

Supplementary Figure 8 (related to Figure 6)

(A) Localization of Hsp70 in NBs and N-P inclusions in the minimal system

BSR T7/5 cells were infected with CVS strain (RABV) at a MOI of 1 and fixed at 16h p.i (top row) or co-transfected with plasmids pTit-P and pTit-N (in equimolar concentration) and fixed at 24h post-transfection (bottom row). Confocal analysis was performed after staining with a rabbit polyclonal anti-P antibody (P) followed by incubation with Alexa-488 donkey, and a mouse MAb anti-Hsp70 antibody (Hsp70) followed by incubation with Alexa 568 donkey anti-mouse IgG.

(B) Localization of FAK-GFP in NBs and NB-like structures (N-P inclusions)

BSR T7/5 cells were transfected with plasmid encoding FAK-GFP, then infected at an MOI of 1 for 24 h (top row) or co-transfected with plasmids pTit-P and pTit-N and plasmid encoding FAK-GFP (in equimolar concentrations) (Bottom row). Cells were analyzed by confocal microscopy after staining with a mouse monoclonal anti-N antibody followed by incubation with Alexa 568 anti-mouse IgG.

The scale bars correspond to 15 µm. DAPI was used to stain the nuclei.

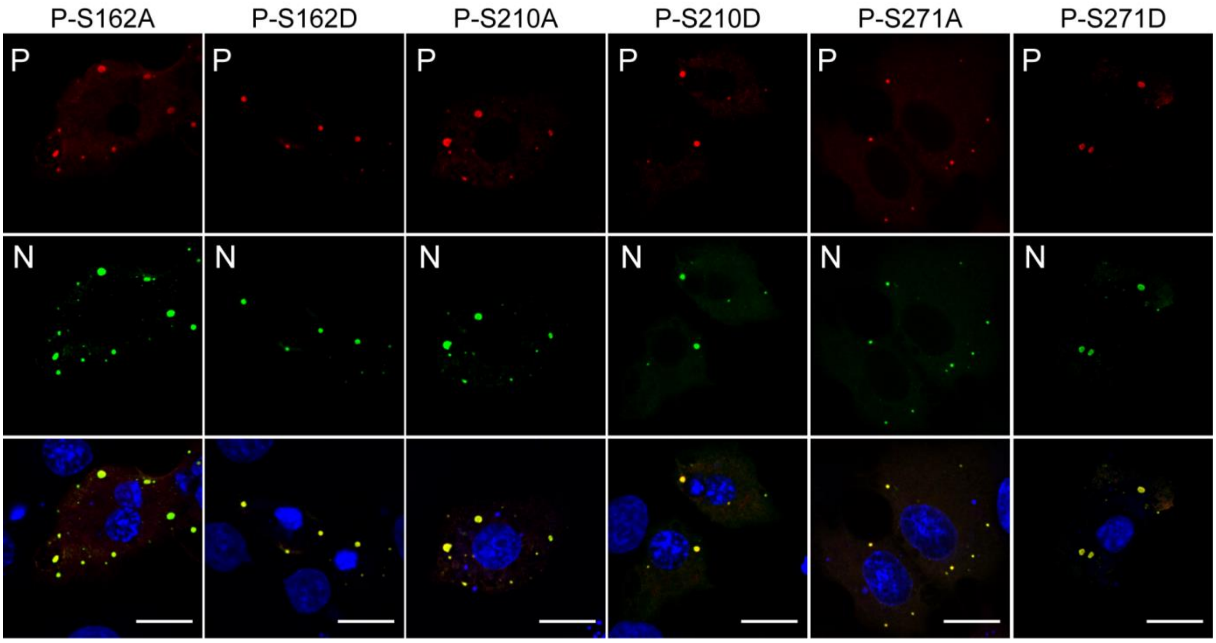

Supplementary Figure 9 (related to Figure 6 D): Role of P phosphorylation in NB-like structures formation

BSR-T7/5 cells were co-transfected for 24h, with plasmids pTit-N and the indicated pTit-P mutants (in equimolar concentration). N was revealed with a mouse monoclonal anti-N antibody followed by incubation with Alexa-488 donkey anti-mouse IgG and P was revealed with a rabbit polyclonal anti-P antibody followed by incubation with Alexa-568 donkey anti-rabbit IgG. DAPI was used to stain the nuclei. Scale bars correspond to 15 µm.

```
                          PNTD P-N0
              *          *          *          *          *          *
CVS    1  MSKIFVNPSAIRAGLADLEMAEETVDLINRNIEDNQAHLQGEPIEVDNLPEDMKRLHLDD
LBV    1  MSKGLIHPSAIRSGLVDLEMAEETVDLVHKNLADSQAHLQGEPLNVDSLPEDMRKMRLTN
MOK    1  MSKDLVHPSLIRAGIVELEMAEETTDLINRTIESNQAHLQGEPLYVDSLPEDMSRLRIED
DUV    1  MSKIFINPSDIRSGLADLEMAEETVELVNRNMEDSQAHLQGVPIDVETLPEDIQRLHITD
EBL1   1  MSKIFVNPSALRSGLADLEMAEETVDLVNKNMEDSQAHLQGIPIDVETLPEDIKRLRIAD
EBL2   1  MSKIFVNPSAIRAGLADLEMAEETVDLVNKNIEDNQAHLQGEPIEVDALPEDMSKLQISE
ABL    1  MSKIFVNPSAIRAGMADLEMAEETVDLINRNIEDNQAHLQGEPIEVDSLPEDIKKLDISE

                    IDD1                                  DD
              *          *          *          *          *          *
CVS   61  EKSSNLGEMVRVGEGKYREDFQMDEGEDPNLLFQSYLDNVGVQIVRQMRSGERFLKIWSQ
LBV   61  APSEREIIEEDEEEYSSEDEYYLSQGQDPMVPFQNFLDELGTQIVRRMKSGDGFFKIWSA
MOK   61  KSRRTKTEEEERDEGSSEEDNYLSEGQDPLIPFQNFLDEIGARAVKRLKTGEGFFRVWSA
DUV   61  PQASLRQDMVDEQKHQEDEDFYLTGRENPLSPFQTHLDAIGLRIVRKMKTGEGFFKIWSQ
EBL1  61  YKQGQREEDASRQEEGEDEDFYMTESENSYVPLQSYLDAVGMQIVRKMKTGDGFFKIWAQ
EBL2  61  RRPAQFTDNTGGKEEGSDEDFYMAESEDPYIPLQSYLEGVGIQLVRQMKTGERFFKIWSQ
ABL   61  GRSKSSADNPQDVDCRMSEDFQMDEVEDPNIQFQSYLDNIGIQIVRKMRTGERFFKIWSQ

                                            IDD2
              *          *          *          *          *          *
CVS  121  TVEEIVSYVTVNFPNPPRRSSEDKSTQTTGRELK-KETTSAFSQRESQPSKARMVAQVAP
LBV  121  ASEDIKGYVLSTFMKPETQATVSKPTQTDSLSVPRPSQGYTSVPRDKPSNSESQGGVKP
MOK  121  LSDDIKGYVSTNIMTSGERDTKSIQIQTEPTASVSSGNESRHDSESMHDPNDKKDHTPDH
DUV  121  AVEDIVSYVALNFSIPVNKLFEDKSTQTVTEKSQQASASSAPNRHEKSSQNARVNSKDAS
EBL1 121  AVEDIVSYVATNFPAPVNKLQADKSTQTTLEKVKQAVSSSAPNKREGPSSNMNLDSQESS
EBL2 121  AVEEIISYVTVHFPMPLGKSTEDKSTQTPEEKFK-PSPQQAVTKKESQSSKIKTISQESS
ABL  121  TVEEIISYVGVNFPNQSGKTTENKSTQTTPKKVK-TEPSSTPAKRSDQLSKTEMAAKTAS

                                                       PCTD P-NARN
              *          *          *          *          *          *
CVS  180  GPPALEWSATNEEDDLSVEAEIAHQIAESFSKKYKFPSRSSGIFLYNFEQLKMNLDDIVK
LBV  181  KKVQKSEWTRDTDEISDIEGEVAHQVAESFSKKYKFPSRSSGIFLWNFEQLKMNLDDIVK
MOK  181  DVVPDIESSTDKGEIRDIEGEVAHQVAESFSKKYKFPSRSSGIFLWNFEQLKMNLDDIVK
DUV  181  GPAALDWTASNEADDESVEAEIAHQIAESFSKKYKFPSRSSGIFLWNFEQLKMNLDEIVR
EBL1 181  GPPGLDWAASNDEDDGSIEAEIAHQIAESFSKKYKFPSRSSGIFLWNFEQLKMNLDDIVR
EBL2 180  GPPALEWSTTNDEENASVEAEIAHQIAESFSKKYKFPSRSSGIFLFNFEQLKMNLDDIVK
ABL  180  GPPALEWPTTNDEDDVSVEAEIAHQIAESFSKKYKFPSRSSGIFLYNFEQLKMNLDDIVK

              *          *          *          *          *          *
CVS  240  EAKNVPGVTRLAHDGSKIPLRCVLGWVALANSKKFQLLVEADKLSKIMQDDLNRYTSC
LBV  241  TSMNVPGVDKIAEKGGKLPLRCILGFVSLDSSKRFRLLADTDKVARLMQDDIHNYMTRIE
MOK  241  AAMNVPGVERIAEKGGKLPLRCILGFVALDSSKRFRLLADNDKVARLIQEDINSYMARLE
DUV  241  EVKEIPGVIKMAKDGMKLPLRCMLGGVASTHSRRFQILVNPEKLGKVMQEDLDKYLTY
EBL1 241  EVKGIPGVTRMARDGMKLPLRCMLGSVASNHSKRFQILVNSAKLGKLMQDDLNRYLAY
EBL2 240  EAKKIPGVVRLAQDGFRLPLRCILGGVGSVNSKKFQLLVNSDKLGKIMQDDLNRYLAY
ABL  240  EAKSVPGVTSLARDGLRLPLRCILGWVGSSHSKKFQLLVGSEKLNKIMQDDLNRYMSC

LBV  301  EIDHN
MOK  301  EAE
```

Supplementary Figure 10 (related to Figure 6C, 6D, 6E and the discussion). Sequence alignment of P proteins of viruses, representative of the 7 genotypes of lyssaviruses. CVS (Rabies virus strain, genotype 1, Swiss-prot: P22363), LBV (Lagos Bat virus, genotype 2,

Swiss-Prot: O56773), MOK (Mokola Virus, genotype 3, Swiss-Prot: P0C569), DUV (Duvenhage virus, genotype 4, Swiss-Prot: O56774), EBL1 (European Bat Lyssavirus 1, genotype 5, GenBank: AAC04587), EBL2 (European Bat Lyssavirus 2, genotype 6, Swiss-Prot: A4UHQ4), ABL (Australian Bat Lyssa virus, genotype 7, Swiss-Prot: Q91RE5). Conserved residues are in cyan boxes whereas similar ones are in yellow (overall aa sequence conservation is 32 %). The black dashes

show gaps. Rectangular boxes delimit protein domains. PNTD P-N^0: N-terminal domain which binds to N^0; IDD1 and IDD2: intrinsically disordered domains; DD: dimerization domain; PCTD P-N^{ARN}: C-terminal domain which binds to RNA-associated N protein. CVS residues 132 to 150 which are required for the phase transition are in bold.

	A_{fast}	k_{fast} (sec^{-1})	$t_{1/2}= ln2/k_{fast}$ (sec)	*Diffusion coefficient associated with the fast phase* ($\mu m^2/s$)	A_{slow}	k_{slow} (sec^{-1})	y_0
**Cytosolic P-mCherry in transfected cells (Fig. 2E)**	*0,5605*	*1,6903*	*0,410*	*~5*	*0,43*	*0,0896*	-7.10^{-4}
**Cytosolic P-mCherry in infected cells (Fig. 2F)**	*0,5933*	*1,7401*	*0,4*	*~5*	*0,3945*	*0,1504*	*0,004*
**P-mCherry in NBs in infected cells (Fig.2G)**	*0,4189*	*0,1343*	*5,2*	*~0,4*	*0,4985*	*0,0109*	*0,0066*
**P-mCherry in inclusions in cells co-expressing N and P (Fig.6B)**	*0,1477*	*0,123*	*5,6*	*~0,36*	*0,5691*	*0,0144*	2.10^{-4}

Supplementary Table 1 (related to Figure 2E, 2G, 2F and 6B) : FRAP parameters obtained by modeling the curves with a double exponential equation.

The FRAP curves were fitted to a double exponential equation:

$$y(t) = y_0 + A_{fast}\left(1 - e^{-k_{fast}t}\right) + A_{slow}\left(1 - e^{-k_{slow}t}\right)$$

The fitted values of the parameters, the deduced characteristic recovery time of the fast phase and a corresponding diffusion coefficient are indicated.
